# Carbohydrate Staple Food Modulates Gut Microbiota of Mongolians in China

**DOI:** 10.3389/fmicb.2017.00484

**Published:** 2017-03-21

**Authors:** Jing Li, Qiangchuan Hou, Jiachao Zhang, Haiyan Xu, Zhihong Sun, Bilige Menghe, Heping Zhang

**Affiliations:** Key Laboratory of Dairy Biotechnology and Engineering, Education Ministry of P. R. China, Department of Food Science and Engineering, Inner Mongolia Agricultural UniversityHohhot, China

**Keywords:** intestinal microbiome, carbohydrateutilization, shotgun-metagenomic-sequencing, Mongolians, staple food

## Abstract

Gut microbiota is a determining factor in human physiological functions and health. It is commonly accepted that diet has a major influence on the gut microbial community, however, the effects of diet is not fully understood. The typical Mongolian diet is characterized by high and frequent consumption of fermented dairy products and red meat, and low level of carbohydrates. In this study, the gut microbiota profile of 26 Mongolians whom consumed wheat, rice and oat as the sole carbohydrate staple food for a week each consecutively was determined. It was observed that changes in staple carbohydrate rapidly (within a week) altered gut microbial community structure and metabolic pathway of the subjects. Wheat and oat favored bifidobacteria (*Bifidobacterium catenulatum, Bifodobacteriumbifidum, Bifidobacterium adolescentis*); whereas rice suppressed bifidobacteria (*Bifidobacterium longum, Bifidobacterium adolescentis*) and wheat suppresses *Lactobaciilus, Ruminococcus* and *Bacteroides*. The study exhibited two gut microbial clustering patterns with the preference of fucosyllactose utilization linking to fucosidase genes (glycoside hydrolase family classifications: GH95 and GH29) encoded by *Bifidobacterium*, and xylan and arabinoxylan utilization linking to xylanase and arabinoxylanase genes encoded by *Bacteroides*. There was also a correlation between *Lactobacillus ruminis* and sialidase, as well as *Butyrivibrio crossotus* and xylanase/xylosidase. Meanwhile, a strong concordance was found between the gastrointestinal bacterial microbiome and the intestinal virome. Present research will contribute to understanding the impacts of the dietary carbohydrate on human gut microbiome, which will ultimately help understand relationships between dietary factor, microbial populations, and the health of global humans.

## Introduction

The sequencing-based assessment of microbial communities in human feces has uncovered a large quantity (>10^14^) and types (>1,000) of microbial species colonizing the human gut. A growing body of research has indicated that the microbial communities are associated with the digestion of dietary macronutrients, production of nutrients and vitamins and maintenance of the host immunity. Thus, the gastrointestinal microbiota can be considered a highly active metabolic organ to complement the human metabolic activities. Wu et al. (2011) found that two enterotypes (‘*Prevotella*-type’ community and ‘*Bacteroides*-type’ community) were defined among human communities in different geographical regions. The ‘*Prevotella*-type’ community and the ‘*Bacteroides*-type’ community were associated with fiber intake and high protein intake, respectively. The dietary fibers, proteins and peptides, which escape digestion by host enzymes in the upper gut, are metabolized by the microbiota in the colon, thus determine the microbiota in the lower reach of the gut and feces (Macfarlane and Macfarlane, 2012). The metabolites, such as short-chain fatty acids, that are produced by gut bacterial fermentation are available to the hostenterocytes and can influence host physiological processes. The balance of the gut microbial community is a major determining factor in human physiological functions.

Many reports have demonstrated determining factors of the gut microbial community, these includehost genotype, diet, age, body-mass-index, disease and use of antibiotic (Yatsunenko et al., 2012). It is commonly accepted that diet has a major influence on the gut microbial community. The structure of the gut microbial community can be changed by long-term consumption of a habitual diet (Ley et al., 2006; Walker et al., 2011; Wu et al., 2011) as well as short-term consumption of certain diets (David et al., 2014). Dietary alteration in the gut microbiota profile could be temporal (David et al., 2014) or irreversible (Sonnenburg et al., 2016).

In China, the dietary habit is significantly different from the western diets: Chinese diet revolves around a plant-based diet, such as cereal, vegetable, and fruit, supplemented with animal-based protein, such as dairy products, seafood and meat. The grain (wheat, rice and naked oats) is generally called staple food and accounts for a high proportion of the daily diet. In different areas of China, people have different dietary habit: the general staple food for people living south of Yangtze River is rice (*Oryza sativa* var.sinica); in the area north of the Yangtze River, people subsist chiefly on wheat (*Triticum* spp.); whereas people in the north of the Yellow River, one of the major staple food is naked oats (*Avena sativa*). The wheat, rice and naked oats contain different content of non-digestible carbohydrates (resistant starch, non-starch polysaccharides and oligosaccharides). For example, the wheat contains about 1.7% (dry matter) of non-digestible carbohydrates, mainly as xylose and arabinose, the naked oats contain 7.2% (dry matter) of arabinose, and β-glucan, whereas rice contains 0.2% non-digestible carbohydrate (Bednar et al., 2001). The different grain intake may have an influence on the gut microbiota. A diet intervention study based on administration of β-glucans showed changes in the gut microbial composition (De Angelis et al., 2015). The effects of carbohydrate staple diet on the gut microbial community have been little studied. A recent study comparing fecal microbiota profile of central Asian (China and Japan) and Southeast Asia (Indonesia and Thailand) speculated that non-digestible carbohydrates and meat consumption are determining dietary factors in the *Bacteroides*-enterotype (Nakayama et al., 2015). It is the aim of this study to investigate the effect of the three staple food grains on the gut microbial communities via diet intervention.

The typical Mongolian diet is characterized by a high and frequent consumption of fermented dairy products and red meat and a low level of the grain intake. The result of the diet intervention based on the type of carbohydrates for Mongolian may be more obvious than that of the Chinese. In our previous study (Sci. Bull. 61(20): 1605–1614), by using the PacBio single molecule real-time sequencing technology, we revealed the changes in intestinal microbiota of 26 Mongolian volunteers based on 16S rRNA variable regions response to staple carbohydrate. The intestinal microbiome was composed of the microbial taxonomy, microbial function and metabolic pathway. So, previous research only focused on the microbial taxonomic level couldn’t meet our further understanding of the interaction between the intestinal microbiome and diet. Accordingly, in present research the shotgun metagenomic sequencing approach was applied to generate huge sequencing reads of microbial whole genomes. Following procedure analysis involving assembling, gene prediction and pathway annotation enabled us to pay more attention to changes in intestinal microbial functional genes and metabolic pathways. Meanwhile, a robust network based on species, metabolic pathway and food nutrition was constructed for helping us understanding the impacts of the dietary carbohydrate on human gut microbiome, which will ultimately help understand relationships between dietary factor, microbial populations, and the health of global humans.

## Materials and Methods

### Study Design and Sample Collection

In present research, 26 Mongolian subjects (22–35 years old) started the three-week experiments on day 1 upon arrival in China. All subjects had no gastrointestinal disorder and did not take any antibiotics for three months before the experiment started and until the end of the experiment. During the experiment, dietary staple food was replaced each week (the first week: wheat, the second week: rice and third week: oat), and the amount and species of food intake for each individual was the same every day. The information was shown in the Supplementary Table S5. And the diet was prepared by a restaurant according to the designed recipes. All volunteers were instructed to eat only the provided foods or allowable beverages (water or unsweetened tea). They also confirmed that no remarkable changes occurred in their diet intake and no medication was taken during the experiment. The fecal samples of each individual in 4 time points (from week 0 to week 3) were collected for further sequencing. The study protocol was approved by the Ethical Committee of the Inner Mongolia Agriculture University (Hohhot, China). Sampling and all subsequent steps described in the Materials and Methods have been conducted in accordance with the approved guidelines. After obtaining the written informed consent, we collected habitual long-term dietary information from all participants using a food frequency questionnaire (Supplementary Table S6). All subjects gave written informed consent in accordance with the Declaration of Helsinki.

### Metagenomic DNA Extraction

The QIAamp^®^ DNA Stool Mini Kit (Qiagen, Hilden, Germany) was used for DNA extraction from the fecal samples. The quality of the metagenomic DNA was assessed by 0.8% agarose gel electrophoresis. All of the DNA samples were stored at –80°C until further processing.

### Shotgun Metagenomic Sequencing and Quality Control

The Illumina HiSeq2500 platform was used for shotgun metagenomic sequencing. Paired-end reads were generated with 100 bp in the forward and reverse directions. The length of each read was trimmed with Sickle. This set of high-quality reads was then used for a further analysis. An average of 10.34 gigabases (Gb) of paired-end reads were obtained for each sample, totalling more than 1 Tb of high-quality data free of host genomic and adaptor contaminants.

### Shotgun Metagenomic Reads, *De novo* Assembly, Gene Prediction, and Construction of the Non-redundant Gene Catalog

The Illumina reads were assembled into contigs using IDBA-UD (Peng et al., 2012) with default parameters. Genes were predicted on the contigs with MetaGeneMark (Zhu et al., 2010). A non-redundant gene catalog was constructed with CD-HIT (Li and Godzik, 2006) using a sequence identity cut-off of 0.95, with a minimum coverage cut-off of 0.9 for the shorter sequences. This catalog contained 1,617,412 microbial genes.

### Computation of Relative Gene Abundance

To assess the abundance of genes profile, reads were aligned to the gene catalog with Bowtie2 (Langmead et al., 2009) using the following parameters: -p 12 -x nt -1 R1.fastq -2 R2.fastq -S R.sam. Then, for any sample N, we calculated the abundance as follows:

Step 1: Calculation of the copy number of each gene:

(1) $${\text{b}}_{i}=\frac{{\text{x}}_{i}}{{\text{L}}_{i}}$$

Step 2: Calculation of the relative abundance of gene i:

(2) $${\text{a}}_{i}=\frac{{\text{b}}_{i}}{\sum {}_{i}b_{i}}$$

*ai*: the relative abundance of gene *i*

*bi*: the copy number of gene *i* from sample *N*

*Li*: the length of gene *i*

*xi*: the number of mapped reads

### Construction of the Taxonomic Profiling

We use MetaPhlAn2 (Segata et al., 2012) to produce organism abundance profiling with default parameters, which relied on about 1 million unique clade-specific marker genes identified from about 17,000 reference genomes. Meanwhile, the metagenomic reads were annotated by the NCBI virus database to build the virome profile.

### Annotation of KEGG, CAZy, and ARGs Database

We aligned the amino acid sequences that were translated from the gene catalog against the proteins/domains to the Kyoto Encyclopedia of Genes and Genomes (KEGG) and antibiotic resistance genes (ARGs) databases using BLASTP (*e*-value ≤ 1e-5 with a bit-score higher than 60). Each protein was assigned to the KEGG ortholog group (KO) or ARG number by the highest scoring annotated hit. CAZymes (Carbohydrate-Active Enzymes) were predicted from amino acid sequences by against to family-specific HMM of CAZymes in dbCAN database (Yin et al., 2012) using Hmmscan program in HMMER 3.0 package (Eddy, 2009).

### KEGG Pathway Analysis

Differentially enriched KO modules and pathways were identified according to the reporterscores (Patil and Nielsen, 2005) from the *Z*-scores of individual KOs. Accordingly, the *Z* adjusted pathway of each KEGG module and pathway was calculated as previously described (Patil and Nielsen, 2005). Then, the *Z* adjusted pathway was used as the final reporter score for evaluating the enrichment of specific pathways or modules. A reporter score of >2.3 (90% confidence according to the normal distribution) could be used as a detection threshold for significantly differentiating pathways.

### Statistical Analysis

All statistical analyses were undertaken using the R software. PCoA and a kernel density distribution analysis were performed using the ade4 (Zapala and Schork, 2006) package of R software. Differential abundance of species, genes and KOs were tested by the Wilcoxon rank sum test, and the significantly different (*p* < 0.01). The heatmap was built in R using the “pheatmap” package. The correlation between the dietary components, metabolic pathways and relative genera were calculated by Spearman’s rank correlation coefficient and visualized by network in Cytoscape (Version 3.2.1).

### Accession Numbers

The sequence data reported in this paper have been deposited in the NCBI database (Metagenomic data: SRP080787).

## Results

### Dynamic Profile of Gut Microbiome in Response to Changes in Staple Food

The fecal samples for all subjects were collected weekly on 4 time points, at week 0, 1, 2, and 3. Subsequently, microbial DNAs from a total of 104 fecal samples were extracted for metagenomic analysis. By employing the shotgun metagenomic sequencing, we obtained more than 1Tiga base (Tb) of pair-end reads (averagely 61,225,132 high-quality reads for each microbiota). The high-quality reads were assembled into contigs, and the genes were predicted onthe contigs. Then, a non-redundant gene catalog including 1,617,412 genes was constructed. Annotated by HMP database, thus taxonomy profile of each sample was obtained. Meanwhile, the reads were mapped to the collective gene catalog to reconstruct sample-specific gene profile, as well as the profiles for the associated KEGG database Orthologs (KO; Supplementary Table S1).

**FIGURE 1:**
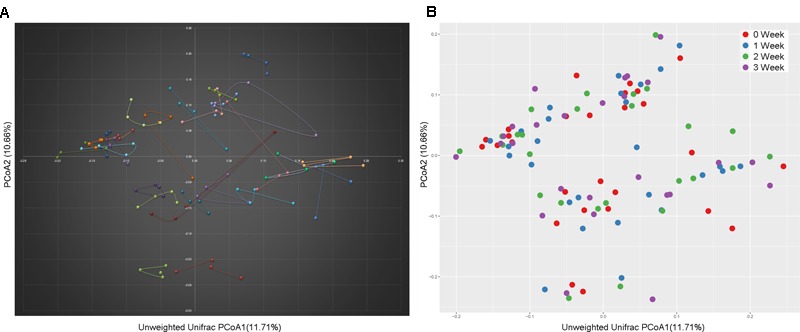
**A principal component (PCoA) score plot based on unweighted UniFrac metrics for all samples. (A)** The sample represented points were connected and colored by individuals; **(B)** the sample represented points were colored by time points.

**FIGURE 2:**
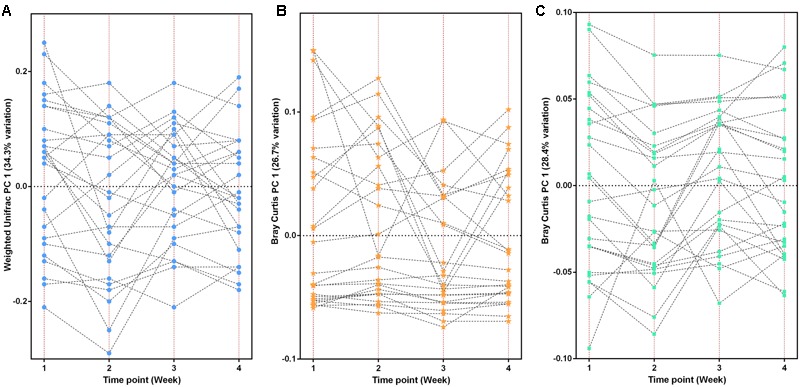
**The changed staple food rapidly altered microbial community structure and metabolic pathway for each individual. (A)** The taxonomic fluctuation of PC1 based on weighted UniFrac matrix. **(B)** The functional (KO features) fluctuation of PC1 based on Bray-Curtis matrix. **(C)** The CAZy fluctuation of PC1 based on Bray–Curtis matrix.

**FIGURE 3:**
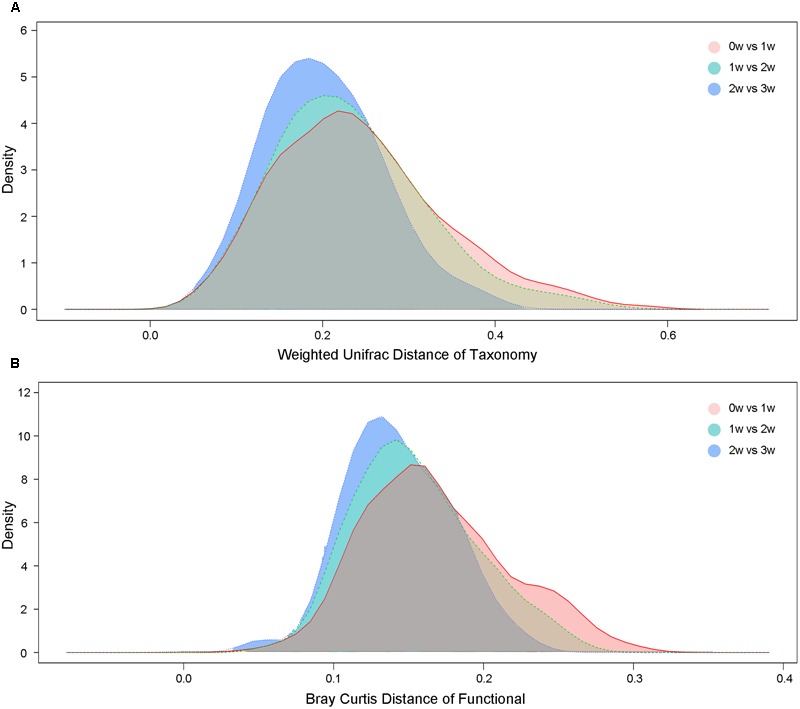
**(A)** The weighted UniFrac distances based on taxonomic abundance among different staple foods groups. **(B)** The Bray–Curtis distances based on functional features abundance among different staple foods groups.

At the taxonomy level, Principal Coordinates Analysis (PCoA) was performed based on the unweighted UniFrac (**Figure 1**), weighted UniFrac (Supplementary Figure S1A) and Bray–Curtis distances (Supplementary Figure S1B) at the species level. No significant apparent clustering pattern was identified among samples in different time points, and the fluctuation in gut microbiota was limited by individuals. It is indicated the staple food-induced change was smaller than the inter-individual difference. This conclusion held true when the data was analyzed at microbial functional feature level (Supplementary Figure S2). By extracting and comparing the first principal component of Bray–Curtis distance matrices representing taxonomic and functional features in gut community structure among subjects at each time point, we found that changed staple food rapidly altered microbial community structure and metabolic pathway (**Figure 2**) for each individual. Then we concluded that the intestinal microbiota could be rapidly altered by staple food, however, the changes was limited at individual’s level. Additionally, in comparing the influence of the three-staple food on gut microbiome, it can be observed from the Weighted UniFrac distance between groups at different time points that the impact of wheat on intestinal microbiota was the largest, followed by rice and oat (**Figure 3**).

**FIGURE 4:**
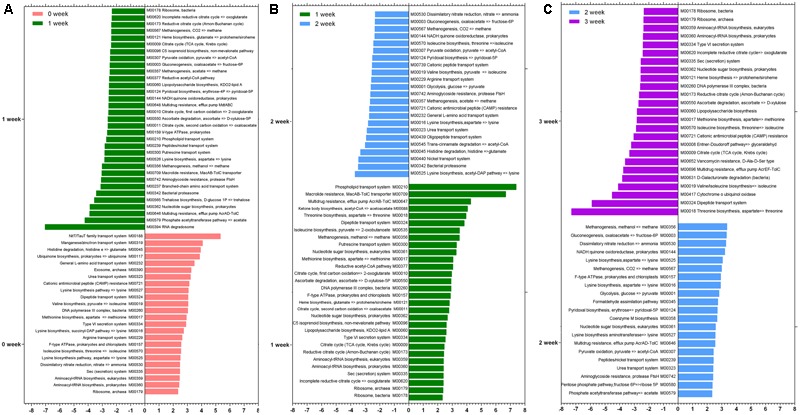
**The reporter *Z* scores of each metabolic module among different staple foods groups by using the reporter feature algorithm. (A)** 0 week vs. 1 week; **(B)** 1 week vs. 2 week; **(C)** 2 week vs. 3 week.

We identified differences in specific species at taxonomy level and metabolic pathway at functional level after the consumption of the respective staple food. As it is shown in **Table 1**, the species *Lactobacillus delbrueckii, Ruminococcus gnavus, Bacteroides vulgatus*, and *Bacteroides massiliensis* decreased significantly but the *Bifidobacterium catenulatum, Bifidobacterium bifidum*, and *Alistipes indistinctus* increased significantly when subjects consumed the wheat as staple food for one week. When rice and oat was assigned as the staple food in the second and third week, respectively, *Bifidobacterium adolescentis, Bifidobacterium longum, Weissella cibaria*, and *Rothia mucilaginosa* declined sharply in the second week whereas the *Bifidobacterium adolescentis* risen sharply in the third week. At the functional level, we calculated the reporter *Z* scores of each pathway or module by using the reporter feature algorithm (**Figure 4**). A reporter score = 2.3 (90% confidence according to the normal distribution) was used as the detection threshold for significantly differentiating modules or pathways. In particular, we found a significant decline in the microbial biosynthesis of amino acids (including valine, leucine, and isoleucine) in the first week. In contrast, the microbial metabolism of carbohydrate kept growing during experiment. Interestingly, it exhibited a high concordance of changes in microbial capacity for fructose metabolism and glycolysis and the changes in relative content of fructose and glucose in wheat, rice and oat. And these results could be confirmed by changes in metabolic models among different staple food intake group.

**Table 1:** Significantly different intestinal species among different time points.

Species	Abundance (%)		Enriched	*P*-value	Species	Abundance (%)		Enriched	*P*-value
**1 week vs. 0 week**	**0 week**	**1 week**	**Enriched**	***P*-value**	*Bifidobacterium catenulatum*	0.616	0.137	1 week	0.0245
*Actinomyces odontolyticus*	0.000	0.001	1 week	0.0360	*Bifidobacterium longum*	2.282	0.482	1 week	0.0004
*Alistipes indistinctus*	0.054	0.148	1 week	0.0300	*Clostridium asparagiforme*	0.005	0.049	2 week	0.0075
*Bacteroides massiliensis*	1.088	0.515	0 week	0.0280	*Coprobacter fastidiosus*	0.008	0.454	2 week	0.0360
*Bacteroide svulgatus*	3.645	1.708	0 week	0.0090	*Dorea formicigenerans*	0.479	0.727	2 week	0.0215
*Bifidobacterium bifidum*	0.373	1.191	1 week	0.0420	*Eubacterium eligens*	0.682	1.635	2 week	0.0049
*Bifidobacterium catenulatum*	0.32	0.616	1 week	0.0210	*Odoribacter splanchnicus*	0.685	1.168	2 week	0.0045
*Eubacterium biforme*	0.242	0.596	1 week	0.0430	*Roseburia hominis*	0.228	0.828	2 week	0.0047
*Eubacterium hallii*	0.015	0.036	1 week	0.0440	*Roseburia inulinivorans*	0.746	1.924	2 week	0.0040
*Lactobacillus delbrueckii*	0.079	0.000	0 week	0.0010	*Rothia mucilaginosa*	0.022	0.008	1 week	0.0361
*Parasutterella excrementihominis*	0.143	0.061	0 week	0.0070	*Ruminococcus torques*	0.698	1.501	2 week	0.0016
*Ruminococcus bromii*	0.408	1.615	1 week	0.0300	*Weissella cibaria*	0.006	0.000	1 week	0.0346
*Ruminococcus gnavus*	0.513	0.078	0 week	0.0010	**3 week vs. 2 week**	**2 week**	**3 week**	**Enriched**	***P*-value**
*Streptococcus parasanguinis*	0.079	0.181	1 week	0.0300	*Anaerotruncus colihominis*	0.016	0.006	2 week	0.0216
*Weissella cibaria*	0.000	0.006	1 week	0.0350	*Bacteroides cellulosilyticus*	0.328	0.179	2 week	0.0086
**2 week vs. 1 week**	**1 week**	**2 week**	**Enriched**	***P*-value**	*Bacteroides thetaiotaomicron*	1.233	0.473	2 week	0.0320
*Anaerostipes hadrus*	0.005	0.01	2 week	0.0365	*Bifidobacterium adolescentis*	1.778	2.843	3 week	0.0321
*Anaerotruncus colihominis*	0.007	0.016	2 week	0.0045	*Clostridium asparagiforme*	0.049	0.017	2 week	0.0382
*Bacteroides cellulosilyticus*	0.109	0.328	2 week	0.0254	*Clostridium leptum*	0.381	0.140	2 week	0.0357
*Bacteroides dorei*	0.336	0.641	2 week	0.0111	*Eubacterium eligens*	1.635	0.799	2 week	0.0074
*Bacteroides intestinalis*	0.028	0.385	2 week	0.0143	*Eubacterium ventriosum*	0.417	0.045	2 week	0.0011
*Bacteroides thetaiotaomicron*	0.412	1.233	2 week	0.0121	*Gordonibacter pamelaeae*	0.041	0.019	2 week	0.0267
*Bacteroides uniformis*	2.233	4.064	2 week	0.0184	*Roseburia hominis*	0.828	0.323	2 week	0.0268
*Bacteroides vulgatus*	1.708	5.207	2 week	0.0001	*Roseburia inulinivorans*	1.924	1.306	2 week	0.0135
*Bifidobacterium adolescentis*	8.286	1.778	1 week	0.0002	*Ruminococcus callidus*	0.512	0.227	2 week	0.0041
*Bifidobacterium angulatum*	0.929	0.308	1 week	0.0185	*Ruminococcus torques*	1.501	0.724	2 week	0.0009
*Bifidobacterium bifidum*	1.191	0.27	1 week	0.0025	*Streptococcus thermophilus*	0.063	0.025	2 week	0.0455

*^∗^Adjusted *P*-values (*P* < 0.05) for the Wilcoxon rank-sum test (paired) are listed.*

### Functional Correlation of Species with Changes in the Staple Food

**FIGURE 5:**
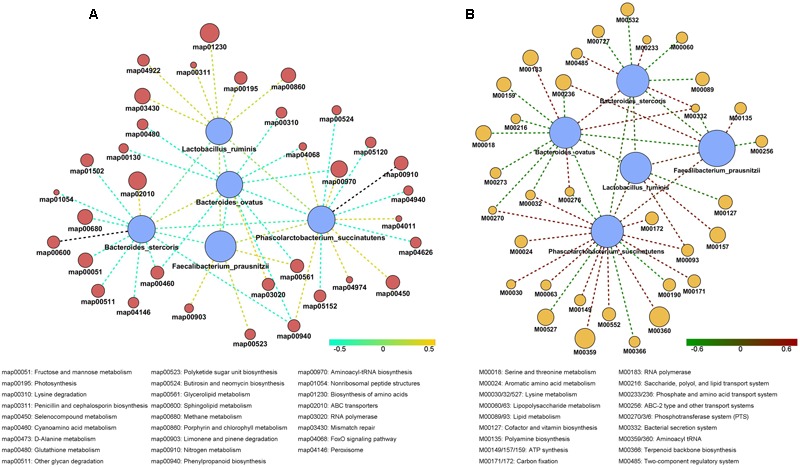
**The correlation among the species in stable content and their typical metabolic pathway (A)** and typical metabolic model **(B)** according to Spearman’s rank correlation coefficient.

Using Wilcoxon sum *t*-test, we identified species which showed significant difference in each staple food group. We focused on the correlation between functionality and changes in the staple food. The species which showed correlations included *Bacteroidesovatus, Faecalibacteriumprausnitzii, Lactobacillus ruminis, Bacteroidesstercoris*, and *Phascolarctobacterium succinatutens*. We determined the correlation among the species and their typical metabolic pathway/model according to Spearman’s rank correlation coefficient (*R* > 0.4, **Figure 5**). Interestingly, a general positive correlation was observed between the species above and the phosphotransferase system (PTS) and the Aminoacyl-tRNA biosynthesis, and a negative correlation with the FoxO signaling pathway and Cyanoamino acid metabolism.

### Concordance of the Gastrointestinal Bacterial Microbiome and Virome

All high-quality sequences were annotated by a virus protein database, and then we selected and constructed the phage or bacterial virus profile. A total of 144 species of phages were identified (Supplementary Table S2), and the quantity of phage of *Enterobacteria, Bacteriophage, Shigella, Klebsiella, Escherichia, Streptococcus, Salmonella, Bacteroides, Lactococcus, Lactobacillus, Clostridium, Haemophilus, Staphylococcus, Brochothrix*, and *Bacillus* accounted for more than 0.001% of the total microbiota (Supplementary Figure S3A). Meanwhile the correlations among the above phages were determined based on Spearman’s rank correlation (Supplementary Figure S3B), and a general positive correlation was found. Additionally, to test the degree of consistency between the observed clustering patterns for bacterial structure and phage structure, respectively, Procrustes analysis was performed based on the PCA matrix of bacterial genus-level organismal profile and that of genus level phage profile (Supplementary Figure S4). The results revealed a strong correspondence between the two profiles above (*P* < 0.001, using 10,000 Monte Carlo label permutations), which also indicated a potential synergistic interaction between the bacteria and bacterial virus in human gastrointestinal tract.

### The Functional Specificity of Intestinal Microbiota for Carbohydrate Utilization

**FIGURE 6:**
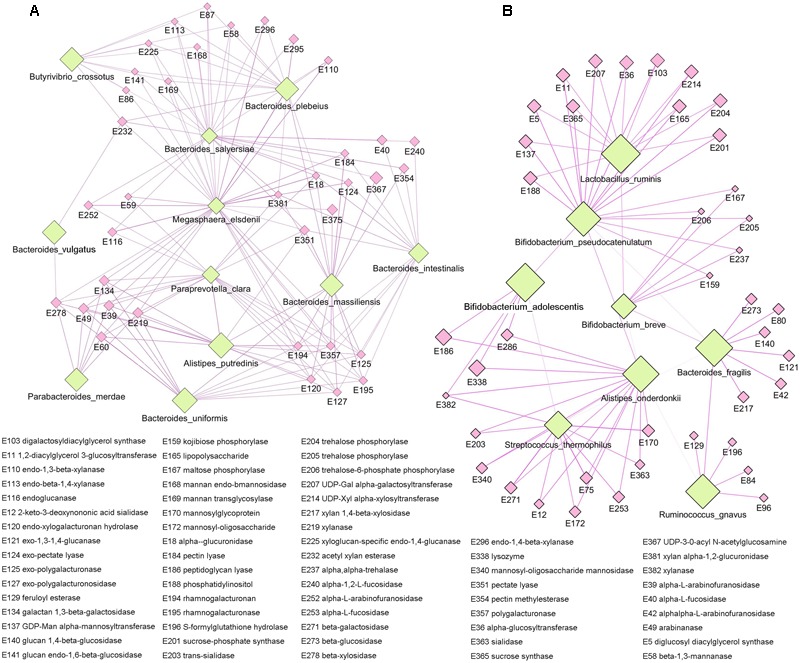
**The specificity of intestinal microbiota for carbohydrate utilizing. (A)** The first species network group were closely related to the carbohydrate-active enzymes of arabinofuranosidase, pectin lyase, polygalacturonase, and xylanase. **(B)** The second species network group were closely related to the carbohydrate-active enzymes of beta-glucosidase, mannosidase, trehalose phosphorylase, fucosidase, and sialidase.

As the main component of the staple food in the present study was carbohydrate, we focused on the functional specificity of intestinal microbiota for carbohydrate utilization. The non-redundant gene catalog in our study was annotated by carbohydrate-active enzymes database (CAZy), and a total of 383 carbohydrate metabolism related enzymes were identified (Supplementary Table S3). By applying the Kruskal-Wallis test, we observed significant difference in enzymes among the different staple food groups, and these enzymes were mainly related to the metabolism of glucose (Supplementary Figure S5). Meanwhile, by clustering analysis, we exhibited the correlation profile between the microbial species and the carbohydrate-active enzymes (Supplementary Figure S6), and two apparent clustering patterns could be found in the profile which indicated two groups of intestinal microbiota with preference for specific carbohydrates utilization. Accordingly, we reconstruct the network between the species and their metabolic enzymes for each group (**Figure 6**). The first group including the species of *Alistipe sputredinis, Bacteroides intestinalis, Bacteroides massiliensis, Bacteroides plebeius, Bacteroides salyersiae, Bacteroides vulgutas, Bacteroides uniformis, Butyrivibrio crossotus, Megaspha eraelsdenii, Parabacteroides merdae*, and *Paraprevotella clara*, and they were closely related to the carbohydrate-active enzymes of arabinofuranosidase, pectin lyase, polygalacturonaseand xylanase. The second group including the species of *Alistipesonderdonkii, Bacteroides fragilis, Bifidobacterium adolescentis, Bifidobacterium breve, Bifidobacterium pseudocatenulatum, Lactobacillus ruminis, Ruminococcus gnavus*, and *Streptococcus thermophilus*, and they were closely related to the carbohydrate-active enzymes of beta-glucosidase, mannosidase, trehalose phosphorylase, fucosidase, and sialidase.

### The Linear Relationship between Intestinal Microbial Phylum and Main Nutrient Elements

**FIGURE 7:**
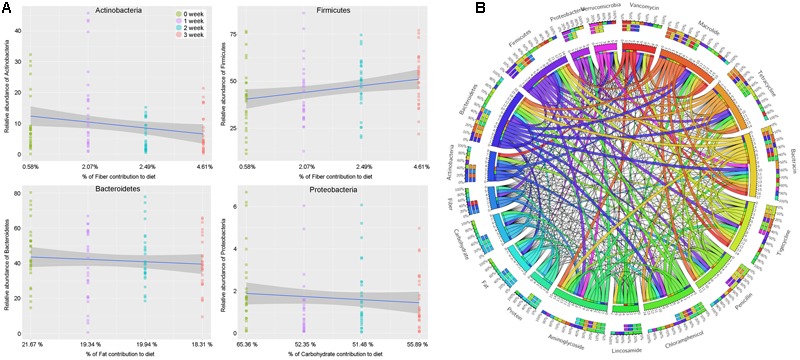
**(A)** The linear relationship between intestinal microbial phylum and main nutrient elements. **(B)** The relationship among the intestinal microbiota, dietary nutrition, and ARGs

The results above based on the impacts of changes in staple food on intestinal microbiome offered us an excellent opportunity to reveal the linear relationship between intestinal microbial phylum and main nutrient elements, which was also the basic mechanism related to the interaction between the diet and gut microbiome. According to the fitted curves, we observed a declined in phylum Actinobacteria but an increase in phylum Firmicutes with the gradually increasing non-digestible carbohydrates (dietary fiber) content in diet (**Figure 7A**). At the same time, the phylum Bacteroidetes and Proteobacteria presented a positive correlation with the dietary fat and digestible carbohydrate, respectively (**Figure 7A**).

### The Relationship between Intestinal Microbiota, Dietary Nutrition, and ARGs

To further understand the dietary effects on human microbiome, we annotated our non-redundant gene catalog with the ARGs database, and constructed the ARGs profile of each individuals. By merging and classifying, 290 annotated ARGs were clustered into 53 antibiotic catalog (Supplementary Table S4). We found that the ARGs related to vancomycin were most abundant, followed by the ARGs related to macrolide, tetracycline, bacitracin, tigecycline, penicillin, chloramphenicol, lincosamide, and aminoglycoside, which gene abundance were more than 0.001%. To describe the relationship among the dietary nutrition, microbial phylum and ARGs above, a Circos on line diagram was constructed (**Figure 7B**).

## Discussion

By employing the shotgun metagenomic sequencing, we described the dynamic profile of gut microbiome in response to changes in staple food. The composition of the gut microbiome can be considered as a complex trait, with the quantitative variation in the microbiome affected by a large number of host and environmental factors, each of which may have only a small additive effect, making it difficult to identify the association for each separate item. A research study of 1135 participants from a Dutch population-based cohort showed relations between the microbiome and 126 exogenous and intrinsic host factors, including 31 intrinsic factors, 12 diseases, 19 drug groups, 4 smoking categories, and 60 dietary factors (Zhernakova et al., 2016). They observed that fecal chromogranin A (CgA), a protein secreted by enteroendocrine cells, was exclusively associated with 61 microbial species whose abundance collectively accounted for 53% of microbial composition. Even so, the diet and the genotype were also considered as the main force for shaping human intestinal microbiome, although it remains unclear if this is primarily driven by host genetics or by extrinsic factors like dietary intake. To address this, another recent study (Carmody et al., 2015) examined the effect of dietary perturbations on the gut microbiota of five inbred mouse strains, and revealed the consumption of a high-fat, high-sugar diet reproducibly altered the gut microbiota despite differences in host genotype. Repeated dietary shifts demonstrated that most changes to the gut microbiota are reversible, which emphasize diet dominates host genotype in shaping the murine gut microbiota. Nevertheless, in the present study, we observed the staple carbohydrate-induced change was much smaller than the inter-individual difference, which indicated the complexity of the interaction between the baseline microbiota and diet change. Besides, we also found that changes in staple food rapidly altered microbial community structure and metabolic pathway for each individual. Recent research also confirmed gut microbiome can rapidly respond to altered diet within 1 day (David et al., 2014), and an animal-based diet had a greater effect on the microbiota than a plant-based diet.

In our previous study, the effect of subjects’ gut microbiota communities by the diet intervention of carbohydrate-rich meals composed principally of wheat, rice and oats was investigated by the PacBio single molecule real-time sequencing technology based on 16S rRNA variable region. During the diet switch within a 3-week period, the bacterial richness and diversity decreased apparently along the diet intervention. And the structure of subjects’ gut microbiota communities after the diet switch was found to be different from those before the diet switch. The previous research only focused on the changes in the microbial taxonomic level, but the intestinal microbiome including the microbial taxonomy and microbial function and metabolic pathway. Accordingly, in present research we pay more attention to the changes in intestinal microbial functional genes and metabolic pathways response to staple carbohydrate. To probe for the potential metabolic mechanism underlying the dominant species (Including *Bacteroides ovatus, Faecalibacterium prausnitzii, Lactobacillus ruminis, Bacteroides stercoris*, an *Phascolarctobacterium succinatutens*) which thrive in the gut environment, we analyzed the functional features in these bacteria, and revealed that these microbes were enriched in the metabolic pathway of PTS. PTS is an active sugar uptake method in low nutrient environment. The bacterial PTS is a perfect example of both the opportunistic nature of bacterial life, and their thrifty use of resources. In the present study, the up-regulation of the metabolic pathway of phosphotransferase efficiently provide the source of energy for the species involved so that they can effectively and stably proliferated in human gut.

The human enteric microbiome contains viruses, bacteria, archaea, fungi, and other eukaryotic organisms (Norman et al., 2014; Virgin, 2014). Enteric human virome and bacterial microbiome alterations have been linked to inflammatory bowel disease (IBD), obesity, and changes in host behavior (Backhed et al., 2012; Lyte, 2013; Norman et al., 2015). In the present study, we observed a robust synergistic interaction between the bacteria and bacterial virus in human gastrointestinal tract in dynamic equilibrium with all components of the microbiome. Accordingly, an emerging concept is that the virome may contain mutualistic symbiotes with some effects that benefit the host and others by modulating the population of their bacterial host. Bacteriophages regulate the bacterial microbiome via gene transfer, killing competing bacteria to allow invasion of prophage-containing bacteria to fill a partly emptied niche, or by encoding toxins that alter the host intestine to foster bacterial pathogenesis (Duerkop and Hooper, 2013).

The ability to utilize complex dietary and host glycans is central to the survival of prominent members of the gut microbiota. Plants in the form of fruits, vegetables, and cereals are major components of the human diet (El Kaoutari et al., 2013b). They provide carbohydrates that are readily digested by human intestinal enzymes, as well as dietary fibers, which are resistant to digestion and absorption in the human small intestine. Carbohydrate-active enzymes encoded by the human gut microbiome catalyze the breakdown of glycoconjugates, oligosaccharides and polysaccharides to fermentable monosaccharides (El Kaoutari et al., 2013a). Here, we exhibited two apparent gut microbial clustering patterns with the preference of specific carbohydrates utilization. For instance, the fucosyllactose utilization was linked tofucosidase genes (glycoside hydrolase family classifications: GH95 and GH29) encoded by *Bifidobacterium* (Milani et al., 2015) and the xylan and arabinoxylan utilization was linked to xylanase and arabinoxylanase genesencoded by *Bacteroides* (Wu et al., 2015). Furthermore, we revealed the robust correlation between the *Lactobacillus ruminis* and sialidase as well as the *Butyrivibriocrossotus* and xylanase/xylosidase. These findings allow modeling of human gut microbiome, responding to diet change, and tailor-designed prebiotics for precision microbiota manipulation.

In summary, this study describes the dynamic profile of gut microbiome in 26 Mongolians in response to changes in staple food (consumed wheat, rice and oat as the sole carbohydrate) for 3 weeks, and revealed the impact of wheat on intestinal microbiota was the largest, followed by rice and oat. Accordingly, we exhibited the specificity of intestinal microbiota for carbohydrate utilizing and the linear relationship between intestinal microbiota and major nutrient elements. Meanwhile, a strong concordance was found between the gastrointestinal bacterial microbiome and the intestinal virome. Present research will contribute to understanding the impacts of the dietary carbohydrate on human gut microbiome, which will ultimately help understand relationships between dietary factor, microbial populations, and the health of global humans.

## Author Contributions

HZ and BM designed the study. JL and HX collected samples. HX and ZS processed and sequenced samples. JZ, QH, and ZS analyzed the data. JZ, JL, and HZ wrote the paper.

## Conflict of Interest Statement

The authors declare that the research was conducted in the absence of any commercial or financial relationships that could be construed as a potential conflict of interest.
