# Supplementary material for: Carbohydrate Staple Food Modulates Gut Microbiota of Mongolians in China
Source: Front Microbiol. 2017 Mar 21;8:484. doi: 10.3389/fmicb.2017.00484 (PMC5359301; doi:10.3389/fmicb.2017.00484)
Supplement: Supplementary file 1 [file Presentation_1.PDF]

Carbohydrate staple food modulates gut microbiota of Mongolians in China

Jing Li*, Qiangchuan Hou*, Jiachao Zhang*, Haiyan Xu, Zhihong Sun, Bilige Menghe†, Heping Zhang†

Key Laboratory of Dairy Biotechnology and Engineering, Education Ministry of P. R. China, Department of Food Science and Engineering, Inner Mongolia Agricultural University, Hohhot 010018, China

*These authors contributed equally to this work.

† Correspondence should be addressed B.M. (email: mhblg@163.com) or H. Z. (hepingdd@vip.sina.com)

The supplementary information including Figure S1 to S6

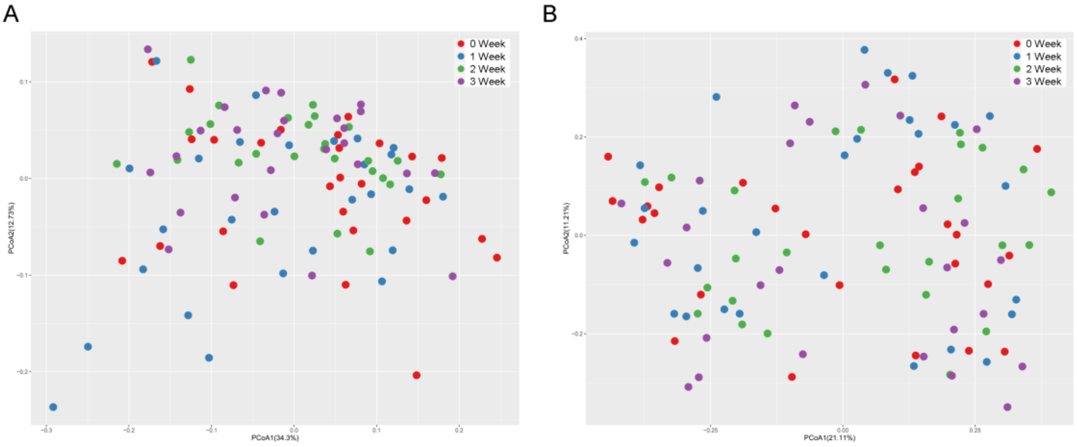

Fig S1 A principal component (PCoA) score plot based on taxonomic metrics for samples in different time points. (A) PCoA score plot constructed based on intestinal microbial relative abundance derived weighted UniFrac distance (different color time points represented samples in different time points) (B) PCoA score plot constructed based on intestinal microbial relative abundance derived Bray Curtis metrics (different color time points represented samples in different time points)

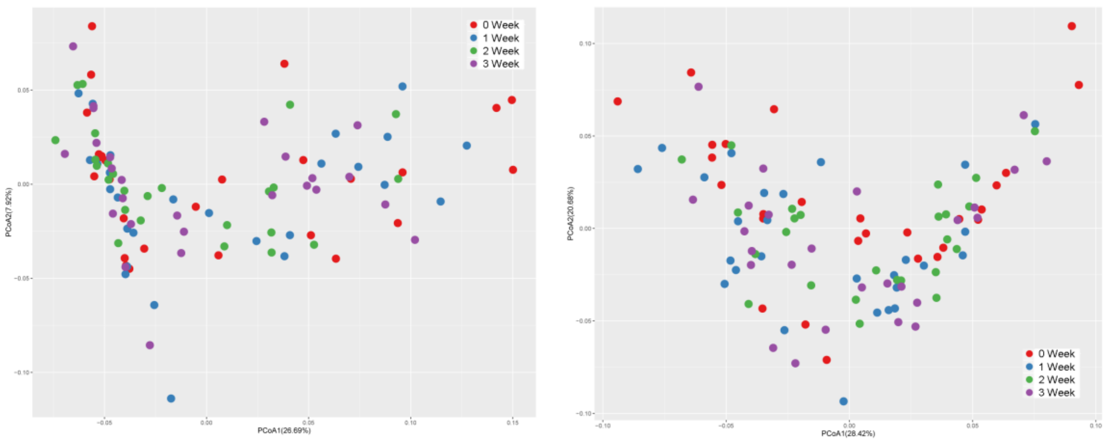

FIG S2 A principal component (PCoA) score plot based on functional metrics for samples in different time points. (A) PCoA score plot constructed based on intestinal functional relative abundance derived weighted UniFrac distance (different color time points represented samples in different time points) (B) PCoA score plot constructed based on intestinal functional relative abundance derived Bray Curtis metrics (different color time points represented samples in different time points)

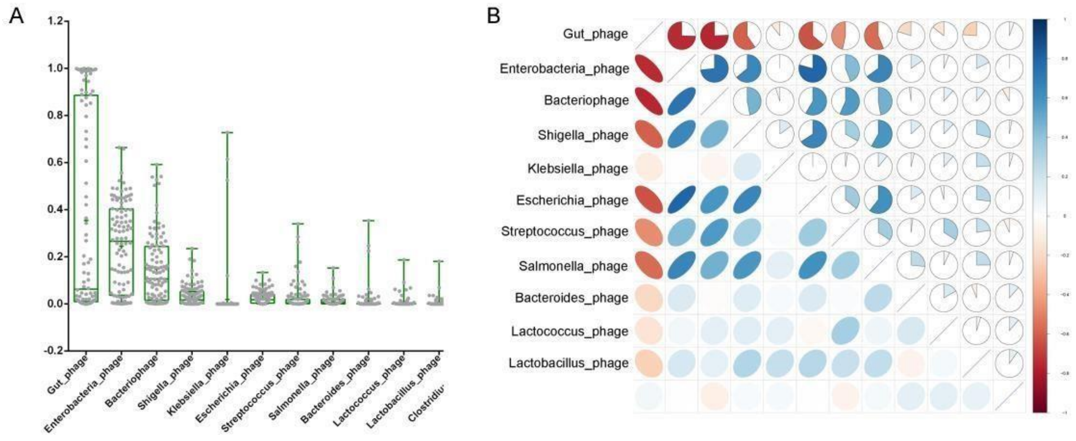

FIG S3 The relative amounts of phages (A) and the correlation between predominant phages (B).

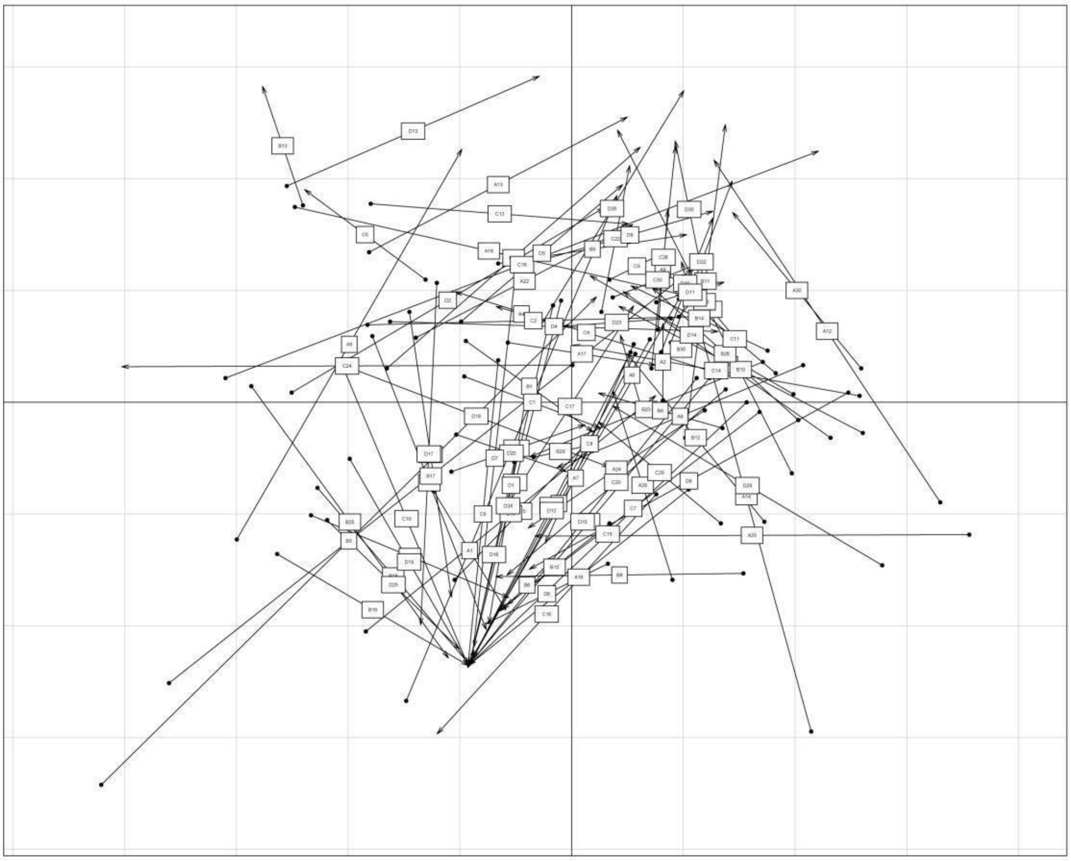

FIG S4 the concordance of the gastrointestinal bacterial microbiome and virome.

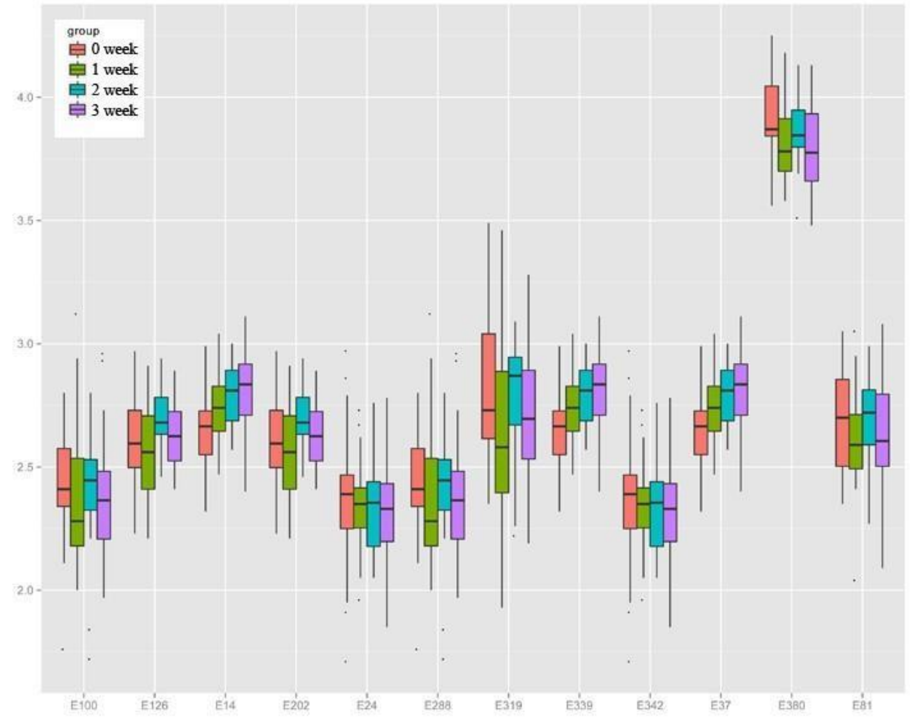

E319 glycosphingolipid beta-N-acetylgalactosaminidase (EC 3.2.1.-) E24 alpha-1,3-glucanase (EC 3.2.1.59)
E342 mycodextranase (EC 3.2.1.61) E14 6-phospho-beta-glucosidase (EC 3.2.1.86)
E37 alpha-glucuronidase (EC 3.2.1.139). E339 maltose-6-phosphate glucosidase (EC 3.2.1.122)
E100 dextranase (EC 3.2.1.11). E288 cycloisomaltooligosaccharide glucanotransferase (EC 2.4.1.-)
E380 unsaturated rhamnogalacturonyl hydrolase (EC 3.2.1.-) E126 exopolygalacturonate lyase (EC 4.2.2.9)
E202 thiopeptidoglycan lyase (EC 4.2.2.-). E81 beta-xylosidase (EC 3.2.1.37)

FIG S5 The significant difference enzymes among the different staple food groups.

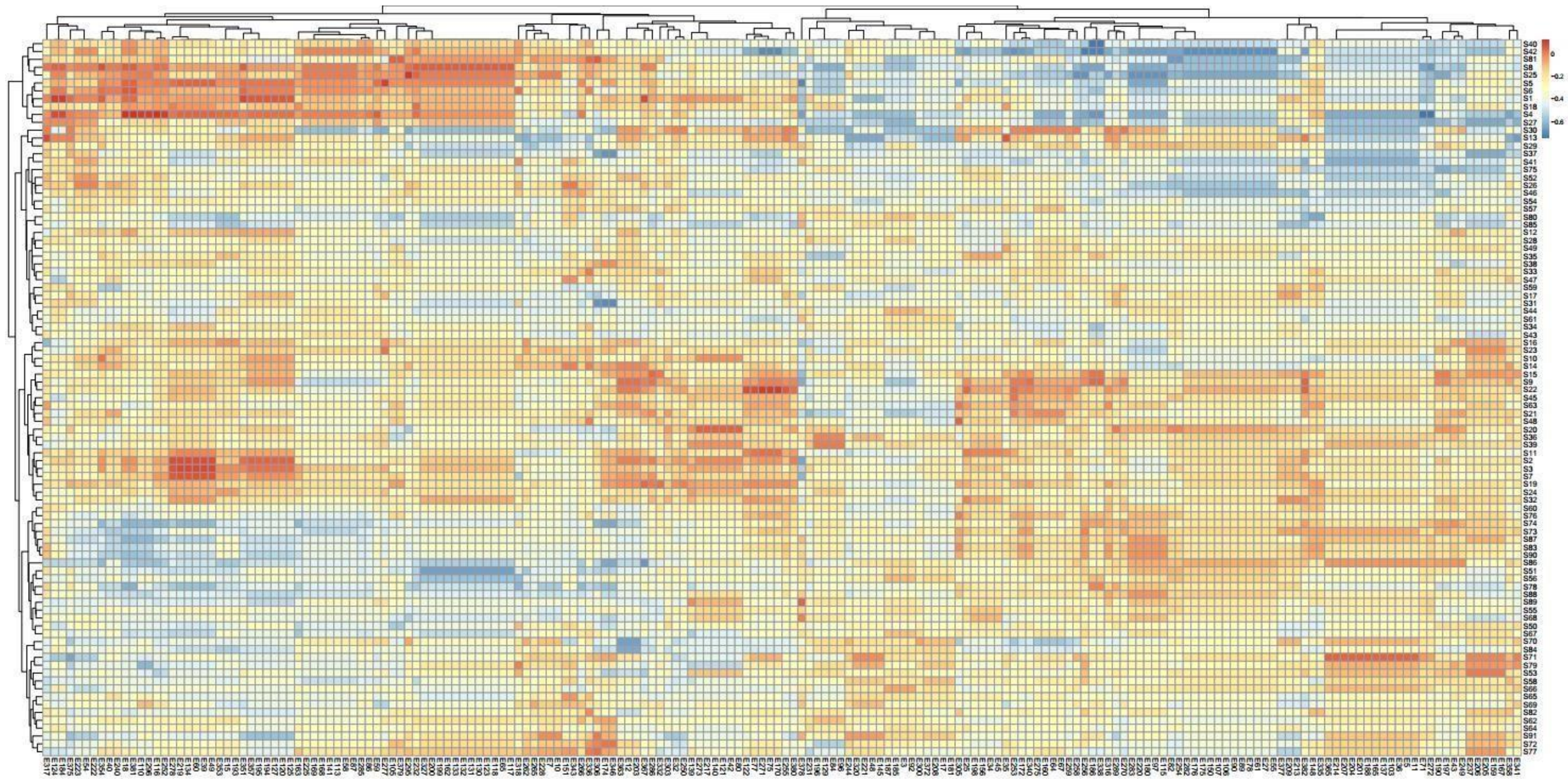

FIG S6 The correlation profile between the microbial species and the carbohydrate-active enzymes.
